# RE: Black ant stings caused by *Pachycondyla sennaarensis*: A significant health hazard

**DOI:** 10.4103/0256-4947.62829

**Published:** 2010

**Authors:** Sulaiman Al Gazlan

**Affiliations:** Department of Medicine, King Faisal Specialist Hospital and Research Centre, Riyadh, Saudi Arabia

**To the Editor:** We read with interest the article on black ant stings by Alanazi et al^1^ and would like to make the following comments. We agree with the authors that black ant stings are a significant and growing problem in Saudi Arabia. Both cases 2 and 3 had significant symptoms after black ant stings. In both cases, among other management, epinephrine was used, but it was given subcutaneously. It should be pointed out that the current recommendation is to give epinephrine intramuscularly in the lateral aspect of the thigh (vastus lateralis)^2^ because there is a significant difference in the absorption of epinephrine when it is given intramuscularly in the lateral aspect of the thigh vs intramuscularly in the deltoid, or subcutaneously in the upper arm. Mean Cmax was significantly higher (*P* <.01) after epinephrine IM injection into the thigh, either from an ampule or an EpiPen, than after epinephrine IM or SC injection into the upper arm, or after saline solution IM or SC injection into the upper arm.^3^

We have a number of patients with black ant allergy and they have tested positive to an extract prepared in house. They carry two pack of Epipen and have been trained in how and when to use them. We are currently in the process of developing immunotherapy for desensitization to black ant venom. We will be very happy to accommodate any patients that need further work up/treatment for black ant allergy.
